# Exergames as an Effective Alternative to Real Environmental Tennis Training for Adolescents

**DOI:** 10.3390/sports12060168

**Published:** 2024-06-17

**Authors:** Fábio Flôres, André Silva, Renata Willig, Ana Reyes, Joana Serpa, Priscila Marconcin, Fernando Vieira, Denise Soares, Nuno Casanova

**Affiliations:** 1 Insight: Piaget Research Center for Ecological Human Development, Piaget Institute, 1950-157 Lisboa, Portugal; fabio.flores@ipiaget.pt (F.F.); renata.willig@ipiaget.pt (R.W.); priscila.marconcin@ipiaget.pt (P.M.);; 2 Research Center in Sports Performance, Recreation, Innovation and Technology (SPRINT), 4960-320 Melgaco, Portugal; 3 Centro de Investigação em Desporto, Educação Física, Exercício e Saúde (CIDEFES), Lusófona University, 1749-024 Lisboa, Portugal; 4 Centro de Investigação, Formação, Inovação e Intervenção em Desporto (CIFI2D), Porto University, 4099-002 Porto, Portugal; 5 Faculty of Health Sciences, Universidad Autónoma de Chile, Providencia 7500912, Chile; 6 Liberal Arts Department, American University of the Middle East, Kuwait City 620000, Kuwait

**Keywords:** reaction time, physical activity, gamification, sports

## Abstract

(1) Background: The popularity of motion-sensing computer-based games, like virtual reality (VR) exergames, is increasing among adolescents. However, their efficacy compared to conventional physical training methods remains unclear. This study investigated whether VR exergames produce effects on reaction time (RT) comparable to traditional tennis training in school-aged adolescents. (2) Methods: In total, 130 adolescents (mean age: 15.6 ± 2.0 years; 67 boys: 15.5 ± 2.2 years; 63 girls: 15.7 ± 1.8 years) were recruited in schools and assigned to one of three groups: VR exergame (G1, n = 39), tennis training (G2, n = 25), or control (G3, n = 66). Participants’ RTs were evaluated before and after the interventions. G1 engaged in VR exergames for 8 min, G2 underwent traditional tennis training for 30 min, and G3 did not participate in any physical activity. (3) Results: Our results indicated that in G3, girls exhibited slower RTs compared to boys (*p* < 0.0). No differences were observed in RTs when comparing G1 and G2. (4) Conclusions: Sex appeared to influence RT, with girls showing slower RTs than boys in G3. The findings suggest that VR exergames and traditional tennis training have similar impacts on RT. This indicates the potential of VR exergames as an alternative to conventional physical training for improving RT in adolescents.

## 1. Introduction

Over the past few decades, there has been an increase in the use of new technologies in our society. The adoption of innovative gadgets and equipment has revolutionized the way people interact, creating additional avenues for social interaction, play, mobility, and exercise [1,2]. This is the case of exergaming, which is the use of computer-based games that use motion-sensing technology that detect users’ (or players’) body movements, allowing for the interaction with and control of different virtual contexts [3]. 

In educational contexts, exergames can offer teachers and students (or players) unique possibilities to diversify physical activity (PA) during physical education (PE) classes and sports participation [4]. Historically, the literature has presented different beliefs about the usage of this new technology, especially in educational settings. Some researchers have argued that television, smartphones, computers, and video games promote sedentary lifestyle patterns [5,6,7]. Nevertheless, the literature has also shown a growing body of evidence indicating that technological advances can provide new ways of practicing exercises and improving PA levels, satisfaction, and learning [1,8,9,10,11,12]. Therefore, these types of virtual games can be more engaging and motivating for players with different skill levels, and they have the potential to increase the duration and intensity of PA [4,13,14,15]. Additionally, exergames can enhance perceptual motor skills, strength, balance, motivation, interest, enjoyment, and engagement among students [1,4]. Huang et al. [8] pointed out that exergaming can positively impact player experience and behavior, as it allows for the combination of entertainment and different kinds of movement. Additionally, some investigations indicate that exergames present positive effects on humor and engagement [16] and in the cardiovascular responses of people with paraplegia [17], as well as changes in health-related behavior [18] and changes in exercise persistence over time [19], while the interactivity and controller use help participants’ physiological and psychological outcomes [20], and they promote enhanced levels of physical fitness [8,21]. However, Ahn et al. [22] showed that a point-based system, such as exergaming, can only briefly increase PA levels, with boys outperforming girls.

Given the high dropout rates from PA these days [23,24,25], one of the most notable benefits of exergaming is its potential to increase the number of individuals who maintain regular PA [26,27]. In fact, Silva et al. [1] showed that exergaming provides the same acute effects in physiological variables as conventional exercises, being a reliable way to improve one’s lifestyle. Moreover, the increase in intrinsic motivation, commitment to the activity, pleasure, and good feelings result in repeated behaviors and maintenance of the PA [28,29]. Since most of these games provide active hand–arm movements, applying great cognitive investment [30], it is also important to consider the impacts of exergaming on motor behavior. Moreover, engaging in exergaming seems to be a promising approach to improving school-aged children’s executive functions [12]. In this sense, reaction time (RT) is a critical component in many physical and cognitive tasks, which can be influenced by a wide range of activities, skills, and participants’ motivation [31,32]. In addition, according to Letovsky [33], hand–eye coordination is very important for RT, and its training could improve RTs among players.

Some investigations have explored the influence of sports and different tasks on the RTs of male and female participants [34,35,36,37,38,39]. For example, Noce and colleagues [40] found that cognitive RT is an important variable in the process of identifying sports talents in tennis. Furthermore, Politopoulos and Tsiatsos [10] proposed an exergame designed to improve the RTs of tennis players. The authors noted that the gaming experience was highly satisfying, according to player feedback, and their results demonstrated that the exergame significantly improved players’ RTs, regardless of their background.

However, there is still a significant gap in research examining the differential impacts of exergaming on RT between sexes. Therefore, the potential of exergames to improve RT warrants further investigation, especially in comparison to traditional training methods. To the best of our knowledge, no prior study has attempted to analyze RTs and compare the effects of exergames with real-world tennis training. The present investigation aimed to determine whether virtual reality (VR) exergames produce similar effects on RT as conventional tennis training sessions in school-aged adolescents. Additionally, we sought to explore potential sex differences in the VR and conventional tennis training groups. We hypothesized that VR exergaming would elicit similar improvements in RT as traditional tennis training, providing a novel context for enhancing this specific skill. Finally, we anticipated that boys would demonstrate better RT results compared to girls.

## 2. Materials and Methods

### 2.1. Participants

The sample size was determined using the GPower v 3.1.9.7 software [41], considering the following parameters: Cohen’s effect size of 0.20 for ANOVA for repeated measures, error probability α = 0.05, and β = 0.95. This calculation indicated a required sample size of at least 102 participants. A total of 130 school-aged adolescents (mean age: 15.6 ± 2.0 years) were conveniently recruited from public and private schools in Portugal, consisting of 67 boys (mean age: 15.5 ± 2.2 years) and 63 girls (mean age: 15.7 ± 1.8 years). Participation was voluntary.

Participants were then randomly assigned to one of three groups: G1—VR exergame (n = 39), G2—tennis training (n = 25), or G3—control (n = 66). The descriptive data are presented in Table 1. None of the adolescents participated in any regular PA programs outside of school, and all had 3 h per week of PE classes.

Inclusion/exclusion criteria were as follows: (a) being within the age range of 10 to 18 years, (b) having no limiting osteoarticular injuries, and (c) not having any illness that would prevent the completion of the study. The study was submitted and approved by the University Ethics Committee (P02-S09-27.04.22) and followed the ethical standards of the Declaration of Helsinki for the study of humans [42].

### 2.2. Procedures

Before data collection commenced, all participants received detailed instructions on how the RT test should be performed. During these instructions, participants remained seated for five minutes without external interference to ensure equal conditions were maintained across the three groups. In addition, all parents signed the written consent form, and all participants verbally agreed to participate.

For the VR exergaming and tennis training groups, each exercise was thoroughly explained and demonstrated to all participants before the practice began. The control group remained in a room for 15 min, during which they were not allowed to engage in any PA or use their smartphones to prevent any interference with the RT measurement.

### 2.3. Measurement and Assessment Tools

#### 2.3.1. Reaction Time Assessment

**Figure 1:**
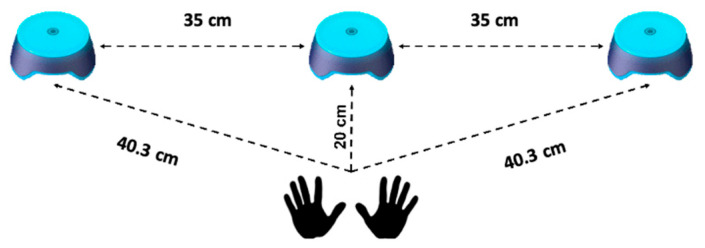
Reaction time test.

The purpose of the test was to measure the time interval between the presentation of a visual stimulus and the participant’s response (in milliseconds). The setup included three pods arranged in a row on a table (35 cm apart, and 20 cm from the pod in the center relative to the participant) (see Figure 1). To perform the task, the participant should sit in front of the pod in the center, with his hands positioned on the table. At the beeping signal, the task starts, and the lights alternate randomly (random time intervals of between 0.5 and 1.5 ms between them). The lights turn off only when the participant presses the pod where the light is on.

Before the start of the test, all adolescents were instructed to press the pods as quickly as possible when the pods lit up. Initially, participants completed a familiarization trial to ensure their understanding of the task. Following the familiarization trial, participants performed a 15 s pre-test with a 20 s interval after the familiarization attempt. Immediately after the end of the exercise (either VR exergaming or tennis training), participants’ RTs were evaluated in the post-test. Throughout the investigation, all trials were conducted using the dominant arm, and no feedback was given to the participants during the tests.

#### 2.3.2. Virtual Reality Exergaming Assessment

We employed the rhythm-based virtual-reality active video game, Beat Saber, which was run on the Meta Quest 2 HMD. In this game, players use the device’s motion-detection controllers to slash at cubes that approach them at various speeds and orientations in sync with the beat of a song. 

Participants played the VR exergaming for 8 min. Research has shown Beat Saber to be a well-tolerated VR experience with minimal aftereffects [43] and rhythm-based games overall to be good training and rehabilitation tools [44].

#### 2.3.3. Tennis Training Assessment

The tennis training assessment involved participants practicing various exercises for 30 min, divided into three 10 min exercises:

- Exercise 1: ball control and perception (10 min).

This exercise is composed of different movements. First, participants hit the ball upwards, letting it hit the ground once before hitting it upwards again. Then, participants hit the ball upwards without letting it fall to the ground. Finally, participants hit the ball downwards without losing control of it (like dribbling).

- Exercise 2: forehand (10 min).

Participants performed forehand movements toward a ball launched over a net.

- Exercise 3: backhand (10 min).

Participants performed backhand movements towards a ball launched over a net.

### 2.4. Data Analysis

Descriptive statistics were used with means and standard deviations for data characterization. The Kolmogorov–Smirnov test was used to verify data normality. RT was used as a dependent variable, and data were analyzed separately according to the following phases: familiarization, pre-test, and post-test. A factorial ANOVA with repeated measures was used to assess the RT scores during the testing phases (familiarization, pre-test, and post-test). The Greenhouse–Geisser adjustment was used to report F values in repeated measures factors [45]. The alpha level of significance was set at 0.05. The software Statistical Package for Social Sciences^TM^ (SPSS 29.0, IBM Corporation, Armonk, NY, USA) was used. 

## 3. Results

The descriptive data for RT for each group are presented in Table 2. Overall, RTs decreased across the phases in both experimental groups.

There were statistically significant differences in RT scores over the investigation phases (F (2, 254) = 22.0, *p* < 0.0; np^2^ = 0.1), *F*(1.9, 235.3) = 46.8, *p* < 0.0. Nevertheless, general comparisons do not show significant differences between the three groups (Tennis—Control, *p* = 0.4; Tennis—VR, *p* = 0.2 (*M*_tennis_ = 463.8, *M*_VR_ = 432.0); and Control—VR, *p* = 1.0). A thorough analysis showed no differences between groups regarding any of the study phases (Table 3). These results showed similar RT results independently of the intervention or control group. The main effect of phase (above) and the main effect of two between-group variables were the following: training exercise, *F*(2, 124) = 3.7, *p* = 0.0 and sex, *F*(1, 124) = 9.7, *p* < 0.0 (*M*_males_ = 430.8, *M*_females_ = 458.4). 

The effect of sex likely influenced the control group only, as post hoc tests showed no significant interaction between group and sex in RTs, except for the control group (in this group, *M*_males_ = 420.2 and *M*_females_ = 455.8, *p* < 0.0). Other post hoc tests are significant, but their main effects or interaction effects are not. One example is the following: in the pre-test, the tennis group has higher RTs than the VR group (*M*_tennisPre_ = 490.5, *M*_VRPre_ = 454.2, *p* = 0.0, *M*_ControlPre_ = 461.4), and in the post-test, the tennis group has higher RTs compared to the control group (*M*_tennisPos_ = 449.7, *M*_ControlPos_ = 417.7, *p* = 0.0), but not the VR group (*M*_VRPos_ = 421.7). So, in the pre-test, the tennis group is slower than the VR group but not more than the control group, and not in the post-test, where it is just slower than the control group. This almost makes it feel like the control group improved more significantly (in fact, the mean differences are tennis = 40.8, VR = 32.5, and control = 43.7). 

Within participants, differences regarding phases can be observed in Table 4, in which none of the groups exhibited significant differences when comparing pre-test with post-test results.

## 4. Discussion

This investigation’s main goal was to assess whether engaging in VR exergames yields comparable effects to conventional tennis training sessions among school-aged adolescents. As our data explore the potential use of VR exergaming as a conventional tennis training exercise, our findings suggest that VR exergaming holds promise as a viable adjunct to conventional training methods in educational and recreational contexts, potentially enhancing overall training performance.

Our main results indicate that there were no significant differences in RT among the three groups (VR exergame, tennis training, and control), suggesting that both VR exergaming and traditional tennis training similarly affect RT. This result challenges the traditional view that training requires direct, real-world interaction to be effective, highlighting the potential of VR exergames as a viable alternative to conventional sports training methodologies to improve RT. For example, Pedersen and colleagues [46] found that exergames were not effective in improving children’s motor skills when compared to PE traditional classes. Despite that, the authors used Nintendo Wii games (tennis contralateral and bowling ipsilateral movements), which are still rudimentary games that, despite emulating the real game, still lack degrees of freedom, unlike what happens in the VR exergame.

Our findings are in accordance with those of Politopoulos and Tsiatsos [10], which showed that exergaming can improve the RT levels of tennis players independently of their sports background. Nevertheless, some caution is needed when interpreting this result. The RT task used in this investigation employs only a computer “click test”, which might not be transferable to real tennis movements. Other similar investigations were found in the literature. For example, Silva et al. [1] found that exergaming exercises produce similar acute physiological effects as conventional training during physical training in young adults. In a systematic review, Mohd Jai et al. [47] suggest that exergames can produce intensity-adequate PA in adults, being beneficial for cardiometabolic improvements. Despite that, the authors highlight that players’ skills and experience levels may contribute to physiological outcomes during exergaming.

Given that RT serves as a metric for measuring information processing in the brain (cognition) [48], our primary findings suggest that a decrease in RT across the phases implies an enhancement in cognitive processing and motor performance. Zeng et al. [12] found that exergaming improves schoolchildren’s executive functions, which refers to a set of cognitive processes that includes working memory, thinking, and self-control, which are crucial for behavior management and achieving tasks, making them vital for learning and development. Moreover, this outcome showed to be a positive result for both exergaming and traditional tennis training, corroborating with previous investigations [1,47,49]. These results challenge the hypothesis that computer-based games are mainly “sedentary tools” that provide prolonged times in sedentary activities.

Contrary to what was expected, another interesting analysis showed that sex differences appear only in the control group, with boys exhibiting faster RTs than girls. This finding implies that the impact of the intervention might vary based on sex [50], although this effect was not seen in the exergaming and tennis training groups [51]. Therefore, the lack of significant changes from the pre-test to the post-test may indicate that both VR exergaming and traditional training impact boys’ and girls’ RTs in the same way. Nevertheless, it is important to highlight that the duration of the tasks (VR exergames and tennis training) was short, which could interfere with our findings. This finding raises some questions about the long-term effects on cognitive performance, and whether other factors, such as exercise intensity or duration, might influence the outcomes. Hence, it is also possible that the investigation’s duration was not sufficient to capture the potential benefits entirely.

Despite our results, our findings are qualified by several limitations. The interventions’ short durations may not be enough to detect the long-term effects of exergaming and traditional tennis training on physical–motor skills performance. Moreover, this investigation did not consider potential dose–response relationships, which could provide valuable insights into the optimal duration and intensity of VR exergaming and traditional tennis training for cognitive benefits. Finally, it was not possible to analyze other variables, such as physiological or mental capabilities, which could improve our analyses and conclusions. 

Further investigations could benefit from a more detailed description of the control group’s activities and should also implement ample time for the intervention. In addition, investigating the effects of the VR exergames during different tasks could provide a better understanding of their influence on RT and other important variables, such as attention, motor competence, or motor learning.

## 5. Conclusions

Our findings indicate no significant difference in RT between VR exergaming and tennis training, suggesting that exergames can effectively mirror the impact of conventional sports training on adolescents. This investigation contributes to the growing body of research on exergaming and its potential impact motor behavior, especially on school-aged adolescents, offering valuable insights into the complex relationship between technology-assisted exercise and cognitive outcomes. Addressing the use of new technologies in different virtual contexts is crucial and should not be overlooked. Finally, even with our promising results, larger trials and samples are needed to confirm our findings.

## Figures and Tables

**Table 1:** Descriptive values of the sample.

Sex	Group	Variables	N	Mean	SD
Boys and Girls	Tennis	Age (y)	25	12.8	1.7
		Weight (kg)	25	47.9	13.6
		Height (m)	25	1.6	0.1
		BMI (kg/m^2^)	25	18.9	2.9
	Virtual reality exergaming	Age (y)	39	16.7	1.2
		Weight (kg)	39	61.7	14.0
		Height (m)	39	1.7	0.1
		BMI (Kg/m^2^)	39	21.5	3.8
	Control	Age (y)	66	16.0	1.6
		Weight (kg)	66	58.9	11.4
		Height (m)	66	1.7	0.1
		BMI (kg/m^2^)	66	21.1	3.2
Boys	Tennis	Age (y)	16	12.7	1.8
		Weight (kg)	16	46.7	13.6
		Height (m)	16	1.6	0.1
		BMI (kg/m^2^)	16	18.6	3.0
	Virtual reality exergaming	Age (y)	18	16.9	1.3
		Weight (kg)	18	68.1	15.1
		Height (m)	18	1.8	0.1
		BMI (kg/m^2^)	18	22.0	3.7
	Control	Age (y)	33	16.1	1.6
		Weight (kg)	33	64.0	12.4
		Height (m)	33	1.7	0.1
		BMI (kg/m^2^)	33	21.5	3.9
Girls	Tennis	Age (y)	9	13.0	1.6
		Weight (kg)	9	50.0	14.1
		Height (m)	9	1.6	0.1
		BMI (kg/m^2^)	9	19.3	2.9
	Virtual reality exergaming	Age (y)	21	16.4	1.2
		Weight (kg)	21	56.3	10.6
		Height (m)	21	1.6	0.1
		BMI (kg/m^2^)	21	21.1	4.0
	Control	Age (y)	33	15.9	1.6
		Weight (kg)	33	53.8	7.4
		Height (m)	33	1.6	0.1
		BMI (kg/m^2^)	33	20.8	2.3

Note: y—years; kg—kilograms; m—meters.

**Table 2:** Descriptive data regarding reaction time.

Group	Phase	N	Minimum	Maximum	Mean	Std. Deviation
Tennis	Familiarization (ms)	25	376	646	493.1	66.9
	Pre-test (ms)	25	351	626	461.4	73.3
	Post-test (ms)	25	339	552	448.4	58.4
Control	Familiarization (ms)	66	358	873	472.3	85.3
	Pre-test (ms)	66	335	637	438.0	63.5
	Post-test (ms)	66	315	776	429.6	77.3
Virtual reality exergaming	Familiarization (ms)	39	361	674	460.1	65.4
	Pre-test (ms)	39	329	715	435.0	77.4
	Post-test (ms)	39	317	520	423.0	49.7

**Table 3:** Pairwise comparisons regarding phases.

Phase	(I) RT	(J) RT	Mean Difference (I–J)	*p*
Familiarization	Tennis	Control	20.9	0.8
		Virtual reality exergaming	33.0	0.3
	Control	Tennis	−20.9	0.7
		Virtual reality exergaming	12.1	1.0
	Virtual reality exergaming	Tennis	−33.0	0.3
		Control	−12.1	1.0
Pre-test	Tennis	Control	23.4	0.5
		Virtual reality exergaming	26.4	0.4
	Control	Tennis	−23.4	0.5
		Virtual reality exergaming	3.1	1.0
	Virtual reality exergaming	Tennis	−26.4	0.4
		Control	−3.1	1.0
Post-test	Tennis	Control	18.9	0.7
		Virtual reality exergaming	25.4	0.4
	Control	Tennis	−18.9	0.7
		Virtual reality exergaming	6.6	1.0
	Virtual reality exergaming	Tennis	−25.4	0.4
		Control	−6.6	1.0

**Table 4:** Pairwise comparisons regarding groups.

Group	(I) RT	(J) RT	Mean Difference (I–J)	Sig
Tennis	Familiarization	Pre-test	31.7	0.1
		Post-test	44.7	0.0
	Pre-test	Familiarization	−31.7	0.1
		Post-test	13.0	0.8
	Post-test	Familiarization	−44.7	0.0
		Pre-test	−13.0	0.8
Control	Familiarization	Pre-test	34.2	0.00
		Post-test	42.7	0.0
	Pre-test	Familiarization	−34.2	0.0
		Post-test	8.5	0.7
	Post-test	Familiarization	−42.7	0.0
		Pre-test	−8.5	0.7
Virtual reality exergaming	Familiarization	Pre-test	25.2	0.1
		Post-test	37.1	0.0
	Pre-test	Familiarization	−25.2	0.1
		Post-test	12.0	0.6
	Post-test	Familiarization	−37.1	0.0
		Pre-test	−12.0	0.6

## Data Availability

No new data were created or analyzed in this study. Data sharing is not applicable to this article.
